# Systemic IL-26 correlates with improved asthma control in children sensitized to dog allergen

**DOI:** 10.1186/s12931-024-02773-7

**Published:** 2024-04-15

**Authors:** Melissa A. Kovach, Ulrika Käck, Karlhans F Che, Bettina Brundin, Jon R. Konradsen, Anders Lindén

**Affiliations:** 1 Unit for Lung and Airway Research, Institute of Environmental Medicine, Karolinska Institutet, Stockholm, 171 77 Sweden; 2 Department of Clinical Science and Education, Södersjukhuset, Karolinska Institutet, Stockholm, Sweden; 3 Sachs´ Children and Youth Hospital, Södersjukhuset, Stockholm, Sweden; 4 Department of Medicine Solna Immunology and Allergy Unit, Karolinska Institutet and Karolinska University Hospital, Stockholm, Sweden; 5 Department of Women’s and Children’s Health, Karolinska Institutet, Stockholm, Sweden; 6 Karolinska Severe COPD Center, Department of Respiratory Medicine and Allergy, Karolinska University Hospital, Stockholm, Sweden

## Abstract

**Background:**

Interleukin (IL)-26 is produced by T helper type 17 (Type 17) cells and exerts immunomodulatory plus antimicrobial effects. Previous studies show that local IL-26 concentrations in the airways are higher in patients with uncontrolled than in those with controlled asthma, and that this intriguing cytokine bears biomarker potential. Here, we determined how systemic IL-26 relates to allergen sensitization, asthma severity, and to IL-17 A in children.

**Methods:**

Serum samples were obtained from children with (*n* = 60) and without (*n* = 17) sensitization to dog allergen, and IL-26 and IL-17 A protein concentrations were measured using ELISA. Self-reported history, including medication use and validated symptom-based questionnaire scores, was recorded.

**Results:**

The serum concentrations of IL-26 were enhanced in allergen-sensitized subjects and correlated with those of IL-17 A in a positive manner. However, the IL-26 concentrations did not markedly differ between allergen-sensitized subjects with and without asthma, eczema, allergic rhinitis, or a history of food allergy. Notably, IL-26 concentrations correlated with increasing Asthma Control Test (ACT) scores in a positive manner and with inhaled corticosteroid in a negative manner, amongst sensitized subjects with asthma. Moreover, subjects with asthma requiring ≥ 1 course of oral corticosteroids in the preceding 12 months had decreased IL-26 concentrations.

**Conclusion:**

This study forwards evidence that systemic IL-26, just like IL-17 A, is involved in allergen sensitization among children. The association of systemic IL-26 with improved asthma control is compatible with the cellular sources being recruited into the airways in severe asthma, which supports that this kinocidin bears potential as a biomarker and therapeutic target.

## Background

Asthma is a complex and heterogenous, yet very common lung disease, affecting approximately 14% of all children worldwide [1, 2]. Although both non-pharmacologic and pharmacologic strategies are mainstays of therapy, pharmacologic means of asthma management often provide suboptimal control of symptoms. This has led to considerable investigation into the immune mechanisms underlying asthma, to identify more specific therapeutic targets, particularly for those with symptoms resistant to inhaled therapy with corticosteroids. Yet another rationale for these investigations has been the need for novel biomarkers that may identify sub-groups of patients at risk for poor disease control and/or exacerbations.

Asthma has historically been considered a primarily T-helper type 2 (Type 2) cytokine-driven disorder, characterized by eosinophil-predominant airway inflammation, elevated IgE synthesis, airway hyperreactivity, and mucus hypersecretion [3]. This Type 2 inflammatory phenotype tends to respond well to standard pharmacological interventions, which primarily rely on combined inhaled therapy with corticosteroids and bronchodilators. Additionally, a variety of more specific anti-Type 2 therapies targeting IgE, interleukin (IL)-4, IL-5, and IL-13 have been successful in treating patients with the eosinophil-predominant endotype and severe clinical disease [4–6]. However, there is considerable evidence that up to 60% of patients with severe asthma are partially resistant to corticosteroids and other anti-Type 2 therapies, and these may display a clinically distinct endotype, characterized primarily by neutrophilic airway inflammation and increased Type 1 and Type 17 cytokine expression [4, 7]. Despite the latter, while IL-17 A immunomodulation has been used effectively in the treatment of certain Type 17-driven inflammatory conditions [8], the two trials of anti-IL-17 A therapies published so far have not proven utility in treating patients with moderate-to-severe asthma, possibly due to the lack of stratification of specific immunological endotypes [9, 10]. For this reason, there is an unmet medical need to identify additional molecular targets for treating the Th17 endotype in patients with severe asthma.

The cytokine IL-26 is a 36 kDa homodimeric protein that belongs to the IL-10 family [11]. Although it was originally described as an exclusive Type 17 cytokine, it has since been demonstrated that IL-26 is produced in structural as well as a variety of both myeloid-derived cell types, including both Type 1 and Type 17 cells [11–15]. In the lung, IL-26 is constitutively expressed by bronchial epithelial cells, lung fibroblasts, alveolar macrophages, and CD4^+^ and CD8^+^ T cells [16–19]. At the local level, it acts by stimulating the production of a variety of pro-inflammatory cytokines in alveolar macrophages, including IL-1β, IL-6, IL-8, and TNFα [16], while simultaneously inhibiting the production of the same cytokines from bronchial epithelial cells, thereby indicating an intriguing cell-specific responsiveness to IL-26 [19]. At the systemic level, IL-26 is likely to be expressed primarily by blood neutrophils, for which it enhances chemotaxis while inhibiting chemokinesis, and downregulate enzymes such as myeloperoxidase and elastase [20]. Moreover, IL-26 exerts directs bactericidal activity, and experimental evidence indicates that this is achieved as a direct molecular effect via membrane pore formation and the formation of complexes with extracellular DNA that act as a ligand for Toll-like receptors (TLRs) [20, 21].

Our group has previously reported that IL-26 concentrations in BAL samples from adults with asthma are decreased compared to control subjects without asthma [22]. However, among adults with asthma, we found that bronchoalveolar lavage (BAL) IL-26 levels are clearly higher in those with uncontrolled asthma as compared to those with controlled disease. Similarly, amongst children with asthma, IL-26 concentrations in induced sputum were enhanced in those with uncontrolled disease as compared to those with controlled disease [23]. Conversely, other investigators found that systemic IL-26 levels are increased in adult patients with asthma as compared with non-asthmatic control subjects, regardless of disease severity or atopic status [24]. Additionally, these investigators identified IL-26 single nucleotide polymorphisms (SNPs) associated with either increased or decreased risk of developing asthma. Moreover, in a smaller case-control study, the same investigators demonstrated that IL-26 concentrations are elevated both locally in sputum as well as systemically in a cohort of adult women with severe uncontrolled asthma as compared with non-asthmatic control subjects [5]. Avramenko and colleagues observed that exhaled IL-26 concentrations are enhanced in both non-obese and obese asthmatics compared with control subjects without asthma [25]. Moreover, they observed that obese subjects with asthma have enhanced systemic IL-26 concentrations as compared with control subjects, whereas non-obese subjects with asthma do not have clearly enhanced systemic IL-26 concentrations compared to control subjects without asthma. While there are differences in the populations assessed in these studies, the differences in local and systemic IL-26 concentrations in subjects with asthma suggest that are compartment-specific mechanisms regulating the production of IL-26. Taken together, these previous findings suggest that IL-26 expression is altered in patients with asthma and motivate further study, given the limited conceptual understanding of the role of IL-26 in asthma.

The aim of the current study was to determine how systemic IL-26 concentrations relate to allergen sensitization, asthma severity, and to concentrations of the archetype Type 17 cytokine IL-17 A, in children. To address this, we analyzed a cohort of children with (*n* = 60) and without (*n* = 17) sensitization to dog allergen. Most subjects in the allergen-sensitized group reported one or more clinical manifestations of allergic inflammation, plus asthma. Serum concentrations of IL-26 and IL-17 A were measured and compared to subjective and objective demographic characteristics to test our hypothesis, namely that systemic IL-26 and IL-17 A levels are altered in allergen-sensitized children, particularly in those with asthma.

## Methods

### Study population

Subjects in this study included pediatric patients recruited from outpatient clinics in the Stockholm area as previously described [26]. Consent was obtained from parents of each study subject. Briefly, children ages 10 to 18 years were recruited and sensitization to dog dander was confirmed by either positive skin prick test response (wheal size > 3 mm), serum IgE to dog dander > 0.10 kU_A_/L, or both. Allergen-sensitized subjects were included regardless of a history of clinical allergy symptoms to dog dander. Subjects with impaired lung function due to any cause other than asthma and any completed or current allergen-specific immunotherapy were excluded from the study. Sixty (60) allergen-sensitized children were included in the study group, and a further 17 non-sensitized children without current symptoms of airway allergy were included in the control group. All subjects underwent venous blood sampling for preparation of serum, clinical history and physical examination, dynamic spirometry, and methacholine bronchoprovocation. Clinical characteristics are summarized in Table 1.

**Table 1:** Clinical Characteristics of Study Subjects

Variable	Control Subjects	Allergic Subjects	P value
No. of patients	17	60	
Mean age (± SD)	13.3 (3.0)	13.1 (2.3)	0.813
Gender (M/F)	8/9	39/21	0.181
BMI (± SD)	20.4 (3.4)	19.8 (3.5)	0.331
Allergic manifestations (%)			
Rhinitis	0 (0)	58 (97)	**< 0.0001**
Asthma	0 (0)	51 (85)	**< 0.0001**
Eczema, current or previous	2 (11.8)	38 (63)	**0.0002**
Food Allergy	0 (0)	39 (65)	**< 0.0001**
ACT Score (SD)	25.9 (1.1)	21.4 (3.5)	**< 0.0001**
AQLQ Score (SD)	6.96 (0.1)	6.09 (0.8)	**< 0.0001**
Symptoms	6.97 (0.1)	5.91 (1.0)	**< 0.0001**
Activity Limitation	6.97 (0.1)	6.31 (0.9)	**< 0.0001**
Emotional Function	7 (0)	5.98 (1.3)	**< 0.0001**
FEV_1_% of predicted (SD)	109.0 (14.8)	100.6 (11.3)	**0.041**
Mean IL-26 pg/mL (SEM)	ND	73.88 (12.59)	**< 0.0001**
Mean IL-17 A pg/mL (SEM)	18.33 (9.38)	50.35 (6.79)	**< 0.0001**

SD – Standard Deviation; SEM – Standard Error of the Mean; ND – Not Detectable

### Interviews and standardized questionnaires

All children and their parents were interviewed according to a modified version of the standardized questionnaire used in the Environmental and Childhood Asthma study [27]. The questionnaire included demographic data; family history of allergy and asthma; personal history of asthma, rhinitis, and other allergic manifestations, and medications and health care usage. Subjects were also administered the well-validated Asthma Control Test (ACT) [28, 29] and Asthma Quality of Life Questionnaire (AQLQ) [30].

### Cytokine measurement

IL-17 A was measured from serum using the Human IL-17 A ELISA Development Kit from MabTech AB (Nacka Strand, Sweden), which has an assay range between 4 and 400 pg/mL. IL-26 was measured from serum using the Human IL-26 ELISA kit from Invitrogen (Carlsbad, CA, USA), which has an assay range between 3.3 and 800 pg/mL. Both ELISAs were performed following the manufacturer’s protocol. Undetectable values of either cytokine are expressed by convention as one half of the lower limit of the standard curve throughout this study.

### Statistics

Categorical data were compared using the χ^2^ test. The significance of group comparisons was determined using either the Mann-Whitney test or Kruskal-Wallis test for multiple comparisons, as appropriate. Error bars represent mean ± SEM. Normal Gaussian distribution of continuous variables was determined using the D’Agostino-Pearson omnibus normality test. Correlations between cytokines and continuous variables were determined with either the Pearson correlation coefficient for data with a normal distribution or the nonparametric Spearman correlation for data with a non-Gaussian distribution. For all analyses, *p* < 0.05 was considered significant. All calculations were performed using GraphPad Prism 7.0 (GraphPad Software, San Diego, CA).

## Results

### Baseline demographics

There were no significant differences between the mean ages, genders, or body mass index (BMI) in the subjects with and without allergen-sensitization (Table 1). However, the reported allergic symptoms were clearly lower in the control group of non-sensitized children than in the allergen-sensitized group; none of the children in the control group reported any history of allergic rhinitis as compared with 97% in the allergen-sensitized group. Other allergic manifestations, such as eczema or food allergies, were more common in the allergen-sensitized group (63% vs. 11.8% and 65% vs. 0%, respectively). In the allergen-sensitized group, 85% of subjects had a physician’s diagnosis of asthma, whereas no subjects in the non-sensitized group had asthma. Accordingly, quality of life scores (ACT, AQLQ) and baseline FEV_1_ were lower in the allergen-sensitized group compared to the control group. Data regarding total and dog allergen-specific IgE as well as IgE to cat and horse antigens, allergen co-sensitization, and exposures for this study material has been previously published [31, 32].

### IL-26 and IL-17 A in children with dog allergen sensitization

**Fig. 1:**
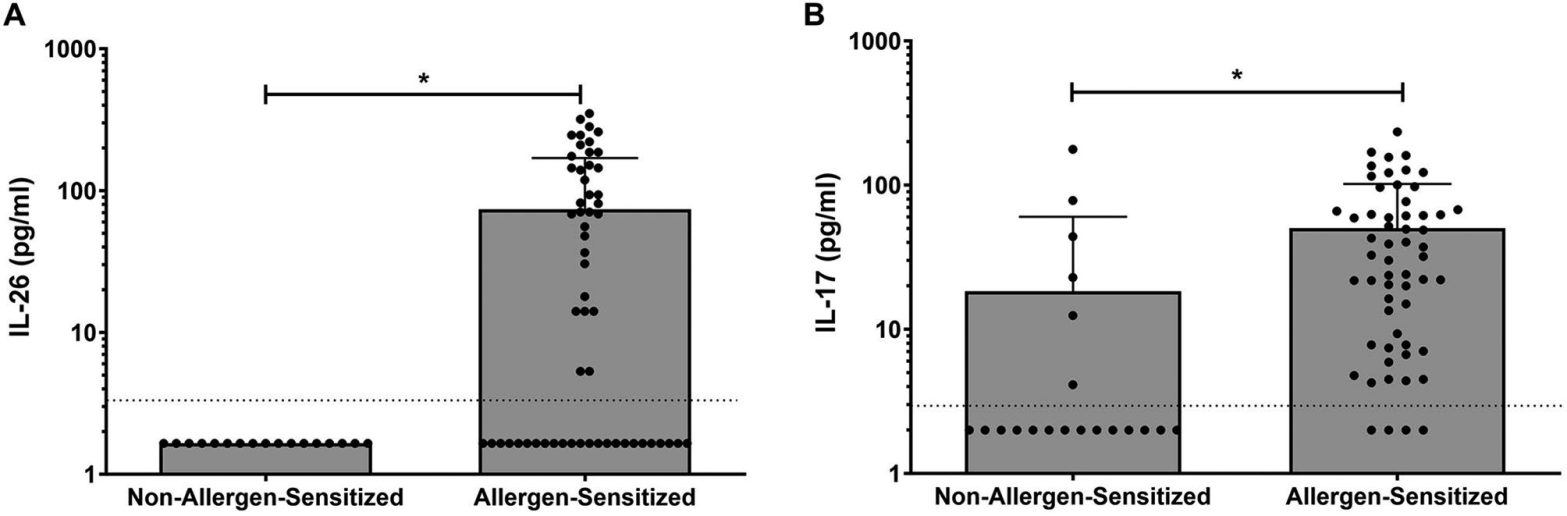
Serum cytokine concentrations in allergen-sensitized subjects vs. controls. IL-26 (**A**) and IL-17 A (**B**) serum concentrations were measured by ELISA in both non-allergen-sensitized and allergen-sensitized study subjects (*n* = 17 and *n* = 60, respectively). Statistical significance was assessed using the Mann-Whitney test. Error bars represent mean ± SEM. Dashed horizonal line represents the lower limit of detection of the ELISA assay

To assess whether release of either of the Type 17 cytokines, IL-26 and IL-17 A, are altered in subjects with confirmed dog allergen sensitization, we measured the protein concentration of each cytokine from frozen serum. Systemic concentrations of both IL-26 and IL-17 A were markedly increased in allergen-sensitized children compared with control children (Fig. 1). No children in the control group had detectable serum IL-26.

### Systemic IL-26 and IL-17 A in children with allergic manifestations

**Fig. 2:**
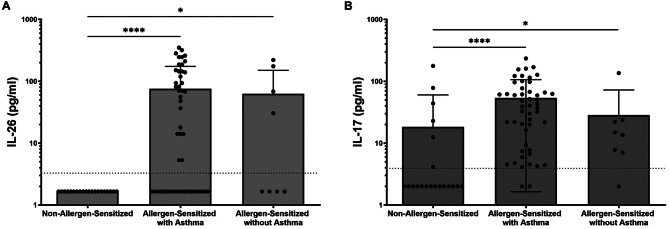
Serum cytokine concentrations in subjects with and without asthma. Serum concentrations of IL-26 (**A**) and IL-17 A (**B**) were measured by ELISA in both non-allergen-sensitized and allergen-sensitized study subjects (*n* = 17 and *n* = 60, respectively). Comparisons were made in subjects with or without asthma. Statistical significance was assessed using the Kruskal-Wallis test for multiple comparisons. Error bars represent mean ± SEM. Dashed horizonal line represents the lower limit of detection of the ELISA assay

Although we observed that both IL-26 and IL-17 A were increased in children with dog allergen sensitization, this does not provide any information about the relative concentrations of each cytokine respective to specific allergic manifestations. To assess this, we compared systemic concentrations of both IL-26 and IL-17 A in those with and without allergic asthma, eczema, allergic rhinitis, or one or more documented food allergies. Children with asthma had significantly higher levels of both IL-26 and IL-17 A (Fig. 2A-B) than children in the control group. Interestingly, serum levels of IL-26 in dog allergen-sensitized children without asthma were also elevated compared with non-sensitized children (Fig. 2A, *p* = 0.023).

**Fig. 3:**
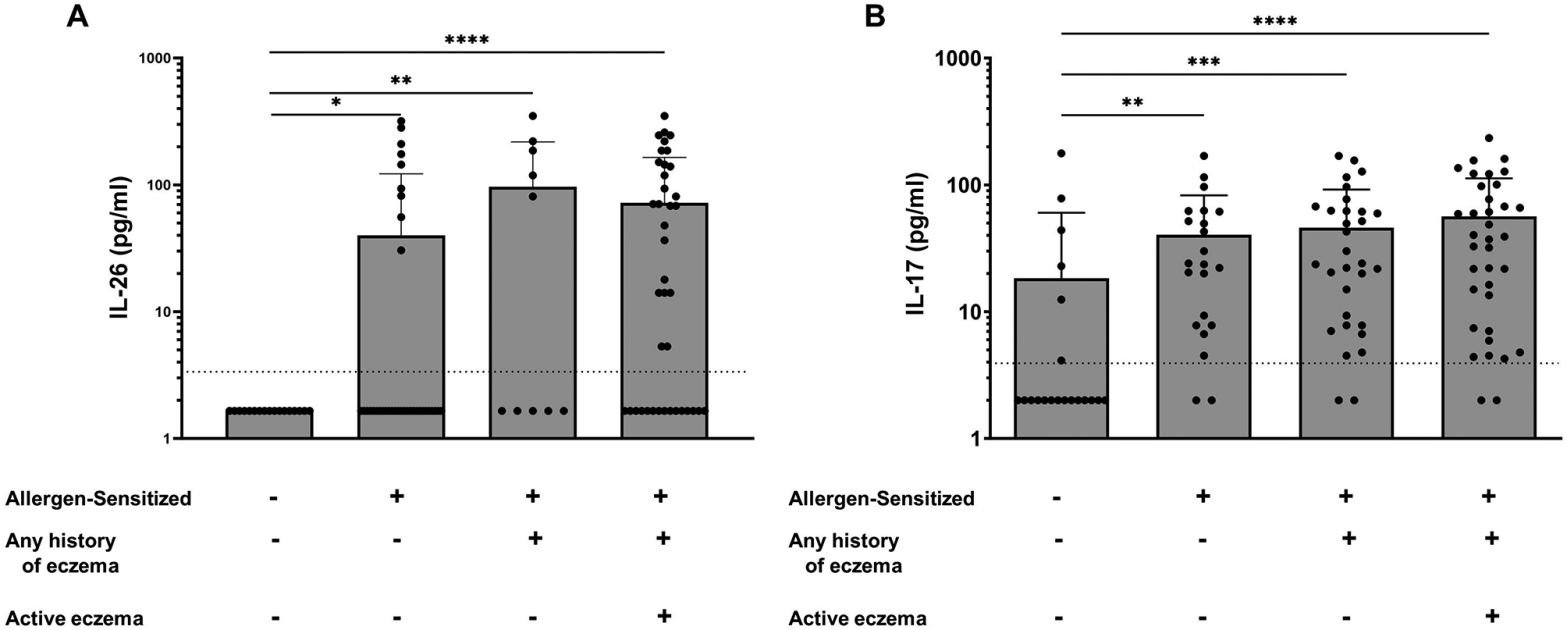
Serum cytokine concentrations in subjects with and without eczema. Serum concentrations of IL-26 (**A**) and IL-17 A (**B**) were measured by ELISA in both non-allergen-sensitized and allergen-sensitized study subjects (*n* = 17 and *n* = 60, respectively). Comparisons were made in subjects with or without eczema. Statistical significance was assessed using the Kruskal-Wallis test for multiple comparisons. Error bars represent mean ± SEM. Dashed horizonal line represents the lower limit of detection of the ELISA assay

**Fig. 4:**
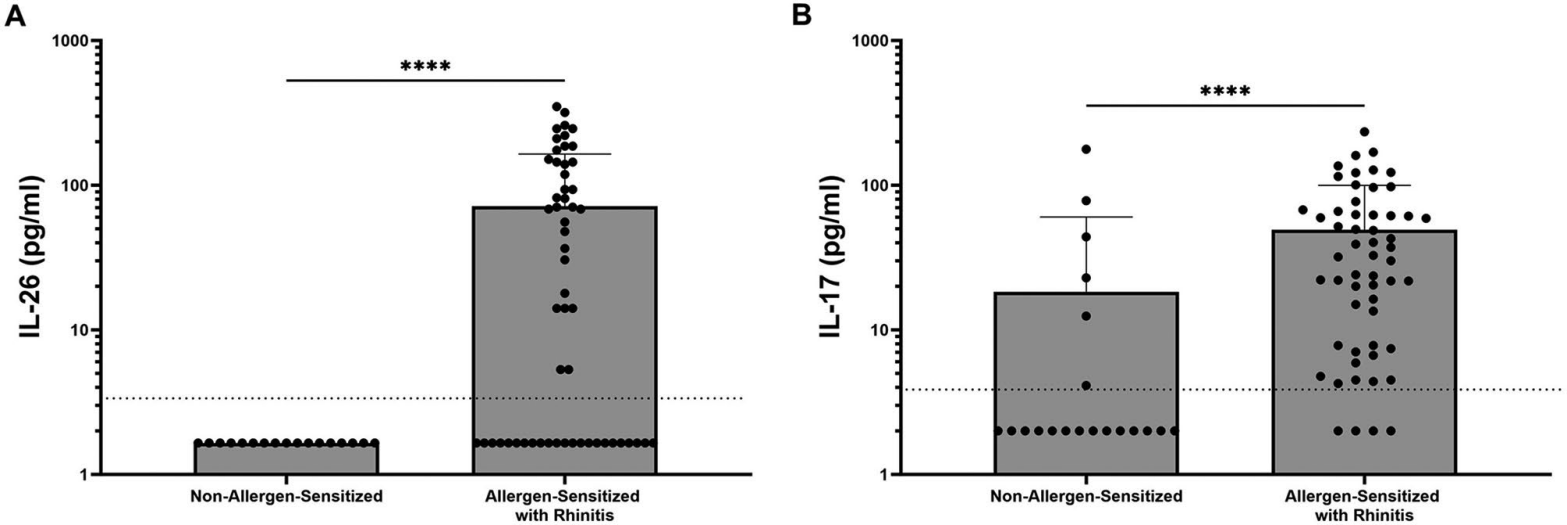
Serum cytokine concentrations in subjects with and without allergic rhinitis. Serum concentrations of IL-26 (**A**) and IL-17 A (**B**) were measured by ELISA in both non-allergen-sensitized and allergen-sensitized study subjects (*n* = 17 and *n* = 60, respectively). Comparisons were made in subjects with or without allergic rhinitis. Statistical significance was assessed using the Kruskal-Wallis test for multiple comparisons. Error bars represent mean ± SEM. Dashed horizonal line represents the lower limit of detection of the ELISA assay

**Fig. 5:**
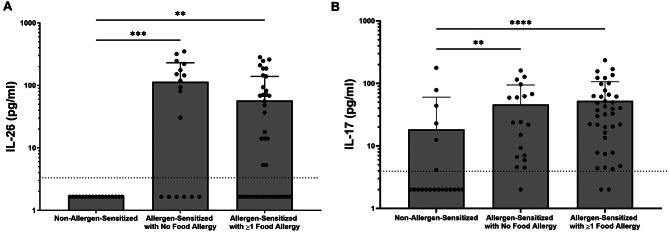
Serum cytokine concentrations in subjects with and without food allergies. Serum concentrations of IL-26 (**A**) and IL-17 A (**B**) were measured by ELISA in both non-allergen-sensitized and allergen-sensitized study subjects (*n* = 17 and *n* = 60, respectively). Comparisons were made in subjects with or without a self-reported history of one or more food allergies. Statistical significance was assessed using the Kruskal-Wallis test for multiple comparisons. Error bars represent mean ± SEM. Dashed horizonal line represents the lower limit of detection of the ELISA assay

Both IL-26 and IL-17 A were also increased systemically in dog allergen-sensitized subjects with eczema compared with control subjects (Fig. 3). This was true for patients with both active eczema as well as those with a history of eczema but without active disease at the time of participation. Additionally, IL-26 and IL-17 A were increased in dog allergen-sensitized subjects with a history of allergic rhinitis (Fig. 4) or a history of at least one previously documented food allergy (Fig. 5).

Of note, given that increasing levels of both IL-26 [33] and IL-17 A [34] in induced sputum have been independently associated with obesity, we assessed each cytokine in normal weight subjects as compared with overweight subjects. There were no subjects with a BMI ≥ 30. No significant difference in either cytokine was observed in overweight subjects, and there were no linear correlations between BMI and either IL-26 or IL-17 A levels (data not shown).

### Systemic IL-26 correlates positively with IL-17 A in allergic children with asthma

**Fig. 6:**
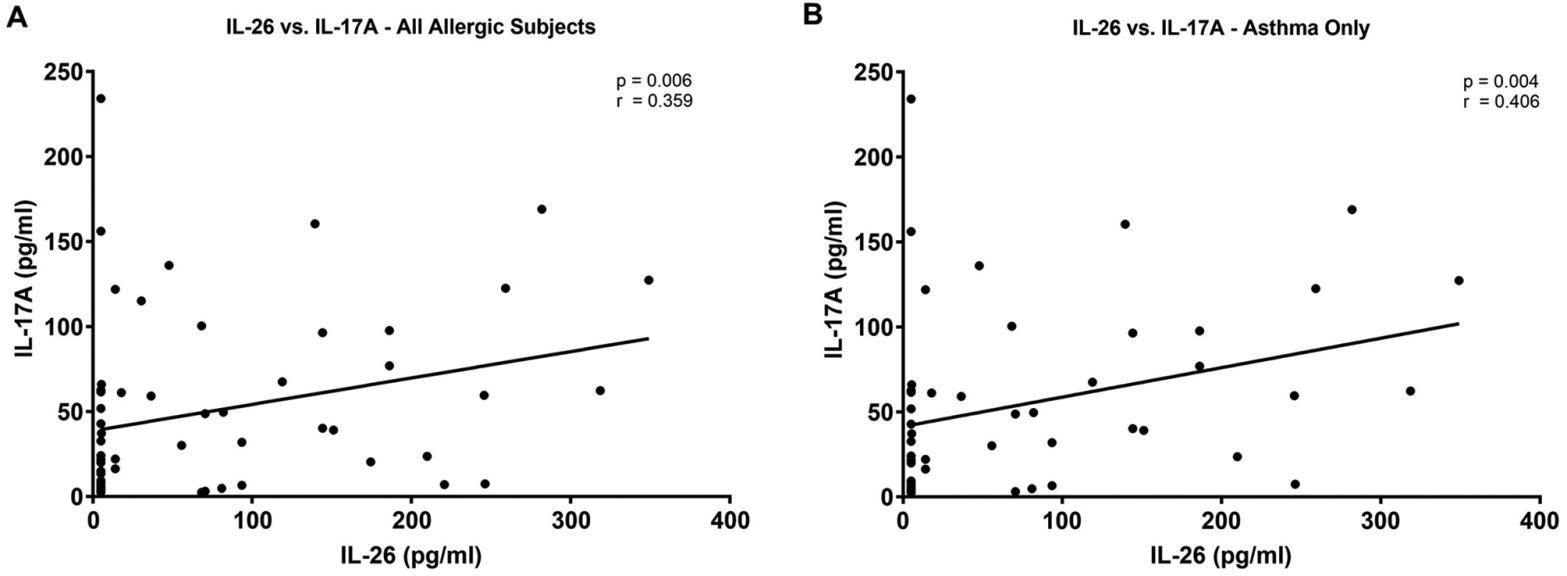
Serum IL-26 concentrations correlate with serum IL-17 A in allergen-sensitized subjects. Serum concentrations of IL-26 and IL-17 A were measured by ELISA in allergen-sensitized subjects. Concentrations of each cytokine were plotted relative to each other for (**A**) all allergen-sensitized subjects (*n* = 60) or (**B**) limited to only allergen-sensitized subjects with asthma (*n* = 51). Statistical significance was assessed using the Spearman correlation coefficient

Having demonstrated that both IL-26 and IL-17 A are elevated systemically in dog allergen-sensitized children compared to non-sensitized children, we next examined whether the systemic concentrations of the two cytokines correlate with each other. Amongst all dog allergen-sensitized subjects, there was a positive correlation between IL-26 and IL-17 A (Fig. 6A). This correlation was also observed in the subset of dog allergen-sensitized children with asthma (Fig. 6B).

### Systemic IL-26 correlates with improved asthma control in allergic children with asthma

**Fig. 7:**
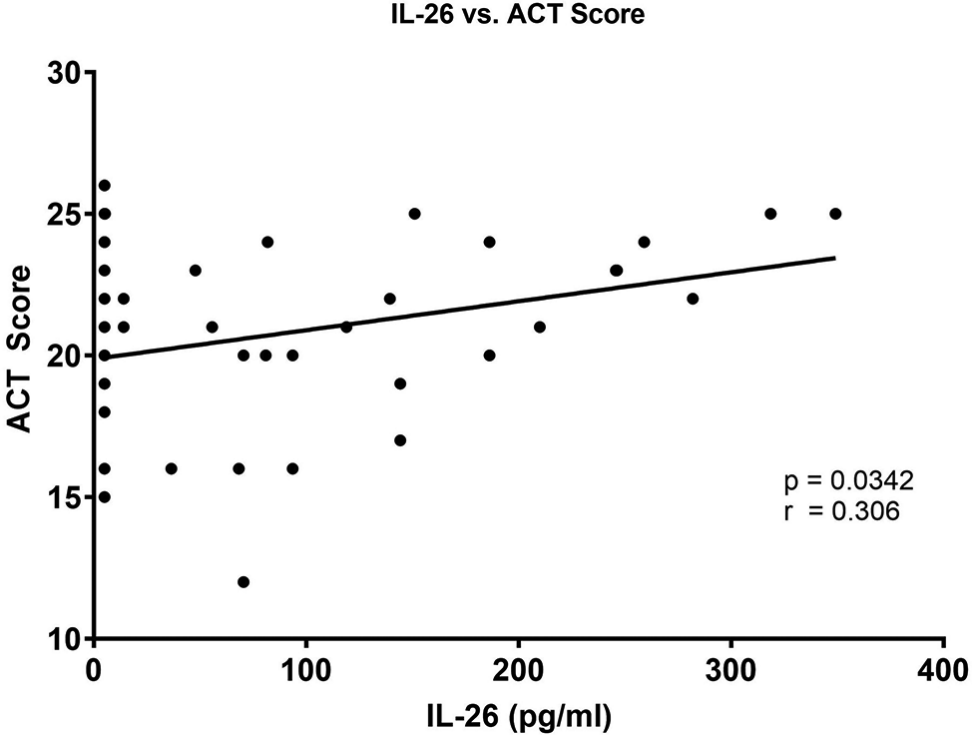
Serum IL-26 concentrations correlate with Asthma Control Test (ACT) Score in allergen-sensitized subjects. Serum concentrations of IL-26 were measured by ELISA in allergen-sensitized subjects with asthma (*n* = 51) and plotted relative to ACT scores. Statistical significance was assessed using the Spearman correlation coefficient

Given that IL-26 is elevated systemically in dog allergen-sensitized children with asthma, we considered whether IL-26 may be a marker of disease severity in asthma. To assess this, we compared the systemic concentration of IL-26 with subjective symptom assessment questionnaires and objective spirometry values. Interestingly, we observed that systemic IL-26 values correlate with increasing, rather than decreasing, ACT scores (Fig. 7). However, we did not find any statistically significant correlations between IL-26 and either AQLQ scores or spirometry values, including FEV_1_ (data not shown).

**Fig. 8:**
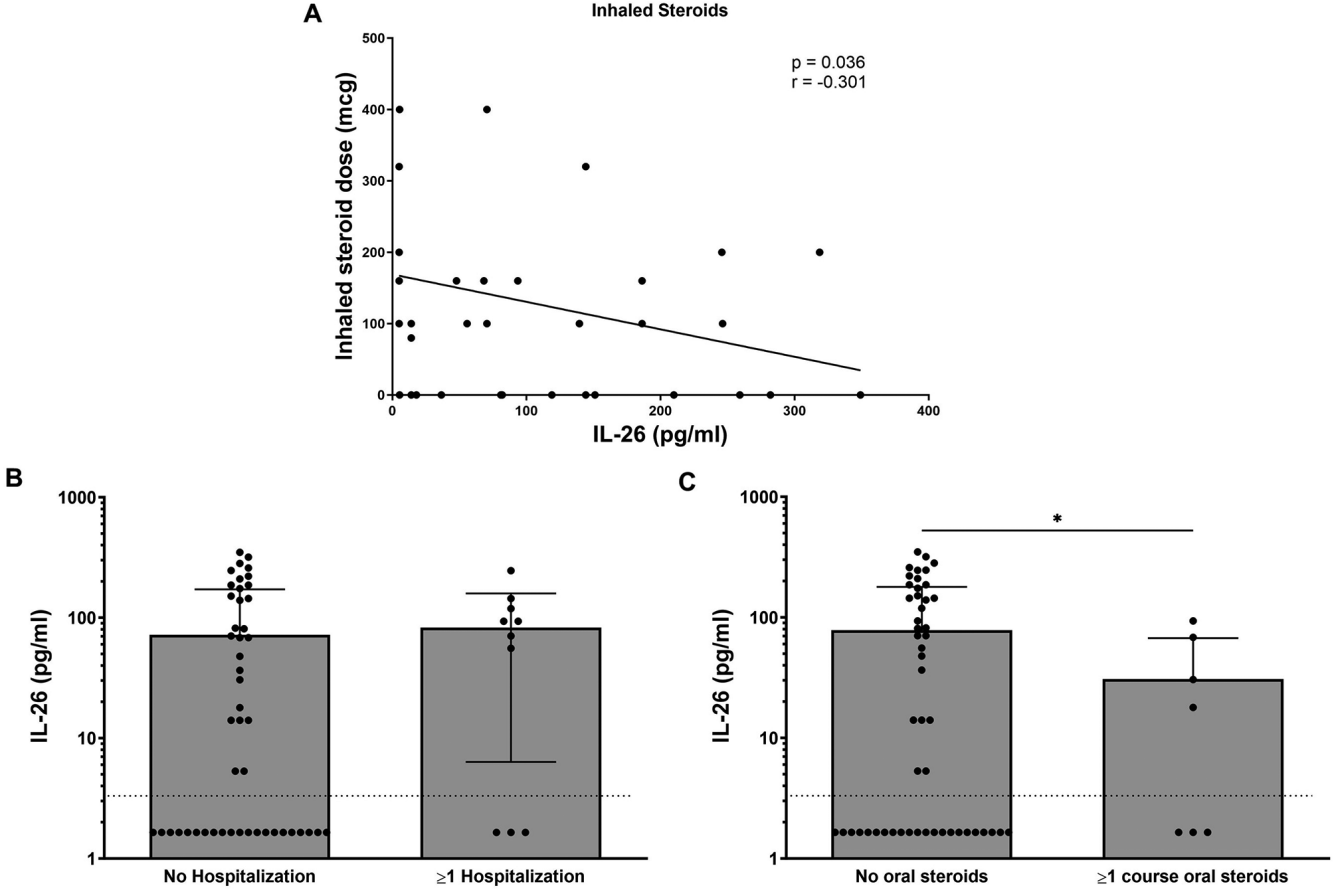
Increasing serum IL-26 concentrations correlate with surrogate markers of asthma severity in allergen-sensitized subjects. Serum concentrations of IL-26 were measured by ELISA in allergen-sensitized subjects with asthma (*n* = 51). (**A**) IL-26 concentrations were plotted relative to each subject’s daily inhaled corticosteroid dose. Statistical significance was assessed using the Spearman correlation coefficient. (**B**) IL-26 concentrations were compared between subjects who had no prior hospitalizations vs. one or more hospitalizations for asthma in the 12 months preceding study enrolment. (**C**) IL-26 concentrations were compared between subjects who had received no oral steroid vs. those receiving one or more courses of oral steroids in the 12 months preceding study enrolment. (**B**-**C**) Statistical significance was assessed using the Mann-Whitney test. Error bars represent mean ± SEM. Dashed horizonal line represents the lower limit of detection of the ELISA assay

Despite a lack of correlation between systemic IL-26 and FEV_1_, we were interested in whether IL-26 levels might also correlate with other objective markers of asthma severity in a similar manner to the subjective correlation to ACT scores. To assess this, we converted each subject’s daily inhaled corticosteroid dose to the equivalent dose of beclomethasone and compared this with their respective systemic IL-26 concentration. We discovered that increasing systemic IL-26 values correlate with decreasing inhaled daily corticosteroid dose (Fig. 8A).

### Previous asthma exacerbations correlate with lower systemic IL-26 levels in allergic children with asthma

Having found that systemic IL-26 concentrations correlate negatively with both ACT scores and daily inhaled corticosteroid use, we also wondered whether there was a significant relationship between systemic IL-26 values and asthma exacerbations. Of note, no subjects were being treated for an active asthma exacerbation at the time of specimen collection. However, information regarding a history of asthma exacerbations in the year leading up to study participation was recorded. Specifically, we assessed the relationship between systemic IL-26 concentrations and two surrogate markers of asthma exacerbations, namely hospitalizations and systemic corticosteroid usage (Fig. 8B-C). We did not find any statistical difference in systemic IL-26 values between subjects that had been hospitalized at least once due to an asthma exacerbation in the year prior to study participation as compared with those who had not been hospitalized (Fig. 8B). However, systemic levels of IL-26 were significantly lower in subjects who had received at least one course of oral corticosteroids to treat an asthma exacerbation in the year preceding study participation as compared to those who had not taken oral corticosteroids (Fig. 8C). No subjects received parenteral corticosteroids in the year prior to study participation.

## Discussion

In the light of the high prevalence of asthma worldwide, particularly given the substantial absolute numbers of patients with severe or “difficult-to-treat” asthma, there is considerable value in identifying new targets that can be utilized as biomarkers, or as targets for novel therapy, thereby facilitating precision medicine. Dog and cat allergen sensitization is relatively common in Europe and elsewhere and is an important trigger for the development of clinical allergic disease, including asthma. The prevalence of both dog and cat sensitization in children is roughly similar in Sweden and Norway across various ages [35]. In this study, we determined whether systemic levels of the Type 17 cytokines IL-26 and IL-17 A are altered in dog allergen-sensitized children, with and without asthma, compared with non-sensitized children. Indeed, we observed that systemic protein concentrations of both IL-26 and IL-17 A are altered in a similar manner; the serum concentrations of both these cytokines are enhanced in children sensitized to dog allergen as compared to non-sensitized children. Based on prior studies demonstrating that systemic concentrations of IL-26 are enhanced in adults with asthma as compared to adults without asthma [5, 24, 25], we were not surprised to discover that both systemic IL-26 and IL-17 A concentrations were likewise enhanced in children sensitized to dog-allergen who also had asthma. However, we were surprised to discover that systemic IL-26 and IL-17 A concentrations did not markedly differ between sensitized children with and without asthma. Moreover, systemic IL-26 and IL-17 A concentrations amongst allergen-sensitized children were not clearly different with respect to other allergic manifestations, namely eczema, allergic rhinitis, or a history of one or more food allergies. In this respect, the presence of elevated IL-26 and/or IL-17 A concentrations in our cohort was not distinctly associated with asthma or specific allergic disease, but rather emerge as general biomarkers of an inflammation that has traditionally been regarded as mediated by Type 2 cytokines. Along with this, we observed a statistically significant positive correlation between systemic IL-26 and IL-17 A concentrations, regardless of whether the analysis included all allergen-sensitized subjects or if it was restricted to only those with asthma. Of note, in the control group of non-sensitized children, there was a small number of subjects with self-reported eczema that did not appear to be associated with elevated levels of either IL-26 or IL-17 A, but it is difficult to make clinically meaningful conclusions from this due to the small sample size. Additionally, although increasing levels of IL-26 [33]and IL-17 A [34] have been associated with obesity independently from the presence or absence of lower airway disease, we did not observe any statistically significant differences in serum levels of either cytokine in subjects with normal as compared to overweight BMI. Furthermore, there was no significant difference in BMI between control and dog allergen-sensitized subjects. It is therefore unlikely that BMI is a confounding variable in our analysis.

Although we found that enhanced IL-26 concentrations are not restricted to children with asthma in our study material, we sought to determine whether IL-26 concentrations may correlate with disease severity amongst allergen-sensitized children with asthma. Previous studies have reported that increasing local IL-26 concentrations correlate with worsening disease severity in adults with asthma [5, 22]. However, Salhi and colleagues did not observe a specific correlation between systemic IL-26 concentration and markers of disease severity in adults with asthma [24]. In our study material, we observed that, amongst children with asthma, increasing systemic IL-26 levels correlate with increasing ACT scores. The higher a patient’s ACT score, the better controlled their asthma symptoms are. Lower scores, particularly scores of 19 or lower, are indicative of poorer control of asthma symptoms. That is to say: We found that increased systemic IL-26 values were associated with improved control of symptoms. However, we detected no statistically significant correlation between systemic IL-26 concentrations and AQLQ scores, nor was there any statistically significant correlation between systemic IL-26 and lung function according to spirometry (FEV_1_, FEV_1_/FVC%) in the current study material. It should be noted however, that the range of spirometry values in the allergen-sensitized study group, while clearly different than the non-sensitized control group, was quite modest and reflects primarily mild-to-moderate asthma. This limited range of disease severity, combined with limited statistical power due to the moderate size of the current material, may explain why we did not detect correlations between systemic IL-26 concentrations and certain clinical assessments.

As these observations were somewhat mixed and were discordant with the previous studies comparing local IL-26 and markers of asthma severity, we wondered whether systemic IL-26 concentrations may correlate with other surrogate markers of asthma severity in a manner similar to that of ACT scores. To this end, we compared systemic IL-26 to daily inhaled corticosteroid doses in the patients with asthma. We observed that increasing systemic IL-26 values correlated with decreasing inhaled corticosteroid doses, which supports the notion that systemic IL-26 concentrations correlate with improved overall symptom control. Moreover, patients that received one or more course of oral corticosteroids for asthma exacerbations in the year preceding study enrolment had significantly lower systemic IL-26 levels compared to the patients with asthma who had not received oral corticosteroids. As patients could not have evidence of an active asthma exacerbation at the time of enrolment, this difference was not due to direct inhibition of systemic IL-26 production by systemic glucocorticoids, a phenomenon which has been described in vitro [18]. If replicable, these findings would suggest that elevated systemic IL-26 concentrations predict milder disease severity in allergen-sensitized children, in contrast with observations of local IL-26 production in the airways. While we have information on serum IL-26 levels only and are unable to speculate further on key sources of production given the many potential sources previously cited by us and others [11–19], our results raise the question of whether these observations indicate that the cellular sources of IL-26 have been recruited into the airways in severe asthma, or whether elevated systemic IL-26 levels per se may confer a protective effect against the development of severe asthma in allergen-sensitized children.

There are some limitations of this retrospective analysis of previously collected study materials. First, the primary focus for patient recruitment was to establish a cohort of allergen-sensitized children, with or without other clinical allergic manifestations. While a majority of these children also had clinically documented asthma, the cohort was not originally designed with this in mind, which could suggest that there are certain differences between our study population and those in similar studies. Second, while the majority of allergen-sensitized children in our study had asthma, most participants had only mild to moderate disease as reflected in spirometry values, ACT and AQLQ scores. This limited range of severity was also reflected by the fact that there were few asthma exacerbations in the 12 months leading up to study enrolment, whereas other studies have focused more on subjects with clinically severe asthma. Nevertheless, it is interesting that so many of the asthmatic subjects in our study had significantly elevated levels of IL-26 and/or IL-17 A, cytokines which are typically associated with more severe and often steroid-resistant asthma. This raises the question of whether the cytokine elevations we observed are merely due to general allergic inflammation in our allergen-sensitized group, or if those children with elevated systemic IL-26 and/or IL-17 A concentrations may be predisposed to developing the more severe Type 1/Type 17 asthma endotype over time. A third limitation of this study is a lack of concurrent airway sampling for analysis, either via sputum or BAL, which would have allowed a concurrent assessment of local and systemic cytokine concentrations, and finally, a lack of concurrent microbiological data. Given that both IL-26 and IL-17 A expression can be affected by microbial pathogens [19, 36, 37], it would be ideal to have a thorough microbiological analysis performed in future studies, to rule out dysbiosis per se as a confounding factor. Finally, given the retrospective nature of this study, the material analyzed was originally powered and collected for scientific purposes other than those in this study, which may limit statistical analysis. However, the robust statistical significance and concordant nature of the findings presented here gives a rationale for a larger, prospective study in order to validate our results and perform statistical estimations of predictive value, sensitivity, and specificity.

## Conclusions

In summary, our findings demonstrate that the systemic levels of both IL-26 and IL-17 A are altered in children with sensitization to dog allergen regardless of clinical allergic symptoms to dog dander, suggesting that both cytokines are involved in allergen sensitization. While elevations of these cytokines are not directly linked to the presence or absence of asthma, increasing systemic IL-26 concentrations are associated with multiple surrogate markers of improved asthma symptom control. Indeed, these observations are compatible with the cellular sources being recruited into the airways in severe asthma, which supports the notion that this kinocidin bears potential both as a biomarker and therapeutic target. Given the clinical relevance of the current observations, our study provides a rationale for larger, prospective studies on the pathogenic role of these Type 17 cytokines, simultaneously examining systemic as well as local cytokine concentrations in asthma, ideally combined with an ex vivo evaluation of key cellular and molecular mechanisms.

## Data Availability

The datasets generated and/or analysed during the current study are not publicly available to protect potentially sensitive or protected patient information but are available from the corresponding author on reasonable request.

## Abbreviations

IL: Interleukin
ELISA: Enzyme-Linked Immunosorbent Assay
ACT: Asthma Control Test
Type 2: T helper Type 2
Type 17: T helper Type 17
TLRs: Toll-like receptors
BAL: Bronchoalveolar lavage
SNPs: Single Nucleotide Polymorphisms
AQLQ: Asthma Quality of Life Questionnaire
BMI: Body Mass Index
FEV_1_: Forced expiratory volume in 1 s
